# Burden and Correlates of Tobacco Use During Pregnancy: Insights From an Urban Resettlement Colony of Delhi

**DOI:** 10.7759/cureus.90444

**Published:** 2025-08-18

**Authors:** Amod Laxmikant Borle, Ajanya V, Shivani Rao, M. Meghachandra Singh

**Affiliations:** 1 Community Medicine, Maulana Azad Medical College, Delhi, IND

**Keywords:** maternal-child health, predictors of tobacco use, prevalence of tobacco use, smokeless tobacco(st), tobacco cessation, tobacco smoke exposure

## Abstract

Introduction

Antenatal tobacco exposure poses significant risks to maternal and fetal health, with implications that extend throughout the life course. Despite these risks, the use of tobacco among pregnant women persists in India, largely driven by sociocultural beliefs, prevailing myths, and inadequate awareness regarding its detrimental effects.

Methods

A community-based cross-sectional study was undertaken among 200 pregnant women residing in an urban resettlement colony of Delhi to estimate the burden, usage patterns, and determinants of tobacco consumption and second-hand smoke (SHS) exposure. Systematic random sampling was employed for participant selection. Data were collected through structured, interviewer-administered questionnaires and analyzed using SPSS version 25 (IBM Corp., Armonk, NY, USA). A *p*-value ≤ 0.05 was considered statistically significant.

Results

The prevalence of current smokeless tobacco (SLT) use was 14 (7%), comprising 10 (5%) less-than-daily and four (2%) daily users. Ghutka was the predominant product used by 12 (85.8%), followed by a combination of Ghutka and Paan. Among daily users, eight (57.1%) reported consumption around four to six times/day, while the rest used it for more than six times/day. Significant associations were observed between current SLT use and sociodemographic variables such as age, educational attainment, employment status, and socioeconomic class. SHS exposure within the household environment was reported by 79 (39.5%) of participants. Public transport emerged as the most frequently observed site of smoking (57.5%), with the lowest frequency reported in educational institutions (0.5%). Awareness regarding the adverse effects of SLT use during pregnancy was limited to 29 (14.5%), and was significantly correlated with age, education level, and socioeconomic status. Notably, none of the SLT users had attempted cessation or received any formal advice or support to quit in the preceding 12 months. Awareness of national cessation services, including the tobacco quitline, was negligible.

Conclusion

The findings reveal a substantial burden of antenatal tobacco exposure, both direct and passive, within vulnerable urban populations, compounded by limited risk perception and absence of cessation support. The integration of tobacco control strategies within routine antenatal care services, coupled with targeted awareness campaigns and behavioral interventions, is imperative to mitigate these preventable risks.

## Introduction

India ranks as the second-largest producer and exporter of tobacco globally [1]. Patterns of tobacco use within the country are shaped by a complex interplay of geographic, sociocultural, economic, and political determinants [2]. Among women, the prevalence of smokeless tobacco (SLT) use remains substantial, attributed to factors such as low cost, cultural acceptability, and the absence of social stigma [3]. Furthermore, widespread misconceptions contribute to its perceived harmlessness, with beliefs that SLT enhances oral and digestive health, alleviates stress, and provides energy for physically demanding labor [4]. According to the Global Adult Tobacco Survey (GATS-2), one in five adults in India uses SLT, while secondary analysis of the National Family Health Survey (NFHS-5) indicates that 2.3% of pregnant women report SLT use [5,6].

Smoke emitted from the burning end of a cigarette or other smoked tobacco products or smoke exhaled by a smoker is defined as secondhand smoke (SHS). No amount of SHS exposure is considered safe, yet 25% of households in India are exposed to SHS daily [7,8].

Tobacco is a major public health problem that annually claims the lives of about 1.35 million people in India. Tobacco consumption imposes a significant socioeconomic burden attributed to the morbidity arising from cancers and various chronic diseases [9]. The vicious cycle of tobacco use among the poor and the exacerbation of poverty due to tobacco-related diseases is well documented [10].

Exposure to tobacco during pregnancy has adverse outcomes for the mother-fetal dyad, not just during pregnancy but throughout the rest of their lives. The effects on pregnant mothers are pre-eclampsia, placenta previa, and placental abruption, while the child has risks of having preterm birth, low birth weight, and stillbirth [11]. Sadly, the knowledge of the adverse health effects of tobacco is deficient even among frontline health workers [12].

This study was carried out with the following objectives: 1) to assess the prevalence, patterns, and sociodemographic correlates of tobacco use among pregnant women of an urban resettlement colony of Delhi; 2) to study the exposure to SHS at home and smoking in different public places as witnessed by participants; 3) to study the awareness regarding adverse effects of antenatal tobacco use in mother and fetus among study participants. 

The study is imperative, as it identifies the perils of tobacco exposure among the vulnerable group of pregnant women residing in the marginalized section of society.

## Materials and methods

A facility-based cross-sectional study was conducted among the women attending the weekly antenatal clinic in the Urban Health Centre of Gokalpuri between July 2023 and January 2024. The centre provides services to approximately 3,383 households with a population of 20,935 residing in an urban resettlement colony. Resettlement colonies are government-planned housing settlements established to accommodate populations displaced by urban development initiatives or relocated from informal settlements.

The calculated sample size turned out to be 194 by considering the prevalence of antenatal tobacco use as 14.8% with an absolute error of 5% at a 95% confidence interval [13].

Women who were residents of the study area for the past six months were included while those requiring urgent referral to a higher center were excluded from the sampling.

Systematic random sampling was done from the new antenatal registrations at the end of each antenatal care session. From the list, the first participant was recruited by simple random sampling (Lottery method) and then the next nine participants were chosen using a sampling interval of two. This process was repeated for 20 consecutive weeks (July to November 2023) till 200 participants were recruited.

A semi-structured questionnaire was developed using the Global Adult Tobacco Survey (GATS) core questionnaire [14]. After written informed consent, sociodemographic details, pattern of tobacco use, exposure to SHS at home, and awareness of adverse effects of antenatal tobacco use were collected by face-to-face interviews. Socioeconomic status was assigned using Modified B G Prasad classification [15]. The pattern of tobacco use encompassed the age of initiation, frequency of use per day, type or form of tobacco used, reason for initiation, and continued use.

Data was analyzed using SPSS 25.0 (IBM Corp., Armonk, NY, USA). Percentages, mean and standard deviation were used for descriptive statistics. Chi-square (χ2) and Fisher's exact test were used in inferential statistics. A p-value of ≤ 0.05 was considered to be statistically significant.

Ethics clearance was taken from the Institutional Ethics Committee of Maulana Azad Medical College, New Delhi (IEC No. F.1/IEC/MAMC/94/06/2022/NO18).

Operational definitions included the following: 1) Current smoker/SLT user: Antenatal women who smoked or used smokeless tobacco, either daily or less than daily, in the past 30 days leading up to the interview, and 2) Past smoker/SLT user: Antenatal women who smoked or used smokeless tobacco, either daily or less than daily, more than 30 days before the interview.

## Results

Sociodemographic characteristics

A total of 200 antenatal women were a part of this study. The ages of women ranged from 18 to 33 years, with a mean age of 25.5 ± 3.2 years. The majority, 191 (95.5%), were Hindus, and the rest were Muslims. Only 78 (39%) had completed high school and 19 (9.5%) were employed. More than half of the participants, 113 (56.5%), belonged to Class III of the Modified BG Prasad scale.

Tobacco use and pattern

**Figure 1:**
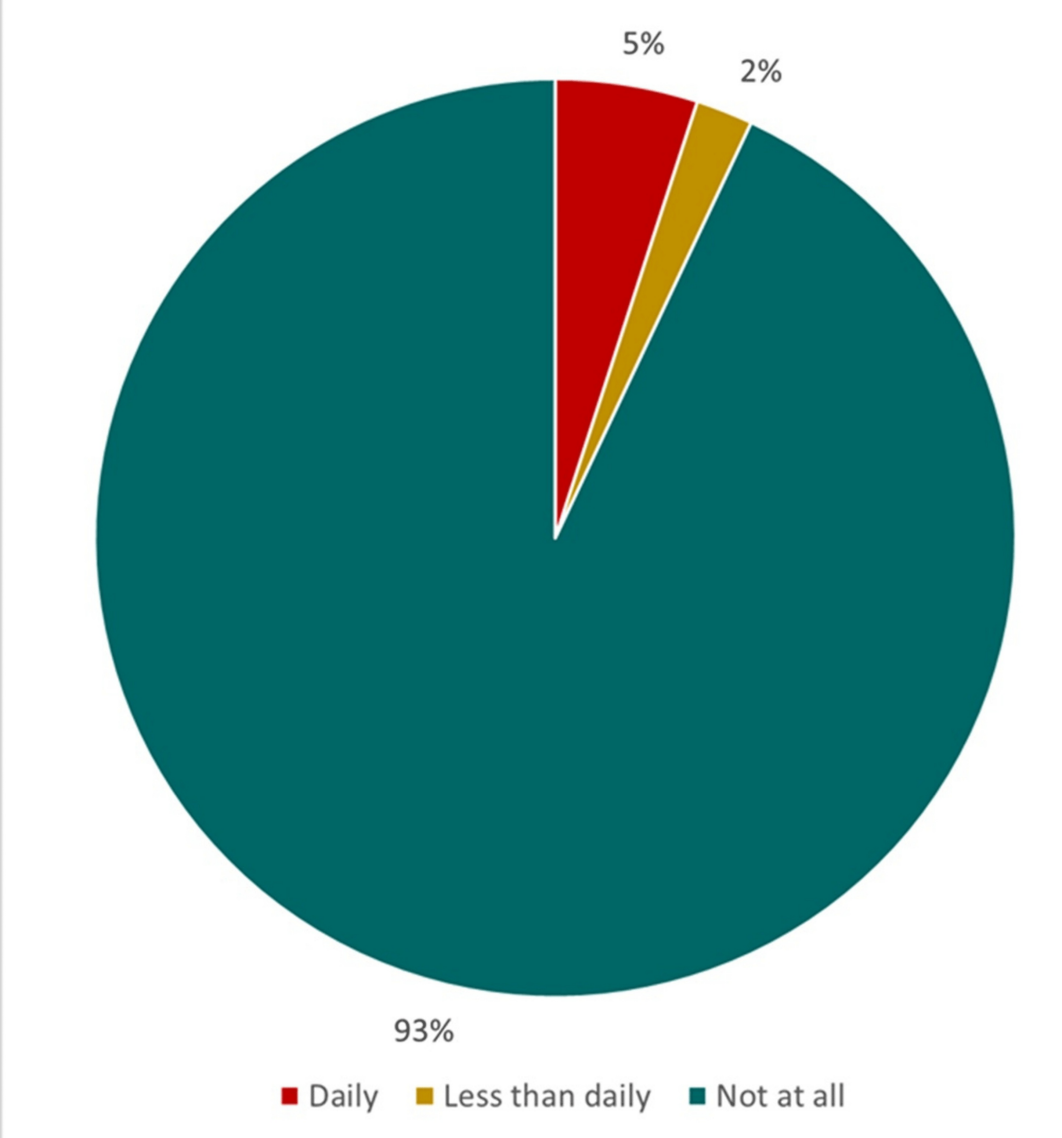
Current smokeless tobacco use (N=200)

Current SLT use among antenatal women was 14 (7%). Current daily users were 10 (5%), and four (2%) were less than daily users (Figure 1). The mean (SD) age of initiation of SLT use was 13.5 (1.9) years. Only Ghutka was used by 12 (85.8%), while two (14.2%) used both Ghutka and Paan. The frequency of daily SLT use was four to six times/day among eight (57.1%) and more than six times/day among the rest. The majority, 10 (71.5%), of current users consumed SLT within 60 minutes of waking up, and four (28.5%) did after. Parental imitation and taste were the reasons for the consumption of SLT. Current SLT use was significantly higher among women aged 26 years and above who have not completed high school, are employed, and belong to lower socioeconomic classes (Table 1).

**Table 1:** Association of sociodemographic factors with current smokeless tobacco (SLT) use using Chi-square test (N=200) #Modified BG Prasad scale [15], * indicates p value ≤0.05

Variables	Current SLT user	Current non user	Chi-square value	p- value
Age groups				0.003*
18 to 25 years	6(4.5)	130(95.5)	4.37	
26 to 33 years	8(12.5)	56(87.5)		
Education				0.049*
Up till Middle school	12(9.9)	110(90.1)	3.86	
High school and above	2(2.6)	76(97.4)		
Type of family				0.744
Nuclear	4(6.1)	61(93.9)	0.10	
Joint	10(7.4)	125(92.6)		
Occupation				0.000*
Unemployed	3(1.7)	178(98.3)	83.53	
Employed	11(57.9)	8(42.1)		
Socio-economic status^#^				0.029*
Class I and II	2(2.4)	82(97.6)	4.74	
Class III and IV	12(10.3)	104(89.7)		

Past daily SLT users were four (2%) and less than daily users were 21 (10.5%). There were no current smokers while past less than daily smokers were six (3%).

Exposure to SHS

**Figure 2:**
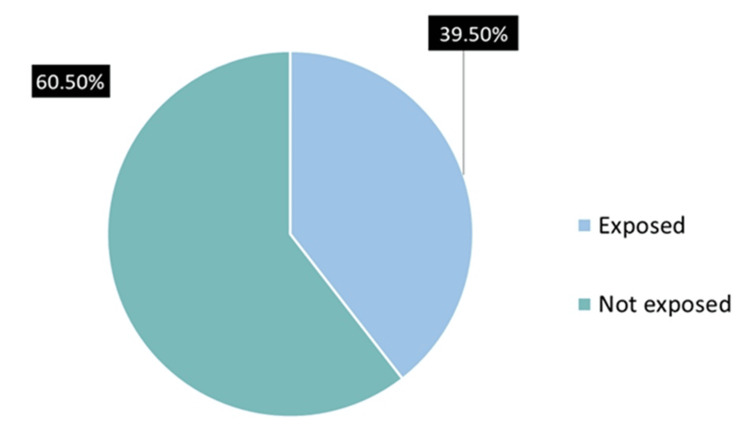
Exposure to second-hand smoke at women's homes (N=200)

**Figure 3:**
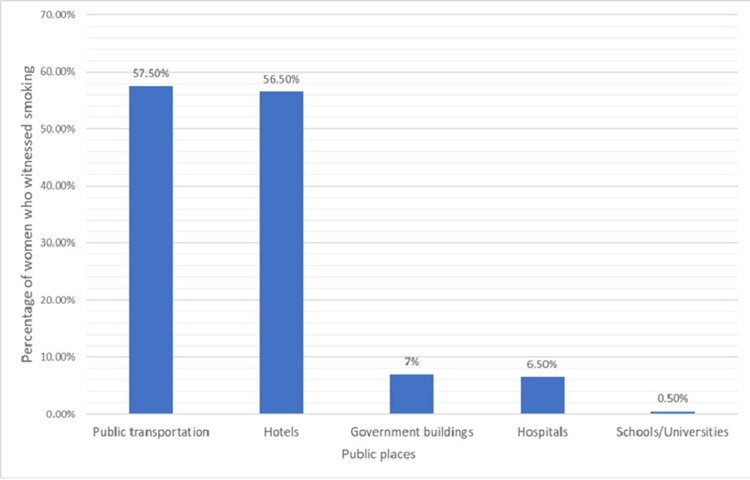
Smoking in public places as witnessed by women (N=200)

Exposure to SHS inside their homes was found in 79 (39.5%) women (Figure 2). Of these, 60 (30%) were exposed daily, four (2%) weekly, two (1%) monthly, and 13 (6.5%) less than monthly. Smoking in public places as witnessed by participants was highest in public transportation (115, 57.5%) and hotels (113, 56.5%), while it was least in schools/universities (one, 0.5%). Smoking in government buildings and hospitals was witnessed by 14 (7%) and 13 (6.5%), respectively (Figure 3). The majority, 156 (78%), of the study participants believed that exposure to SHS doesn’t cause any serious illness.

Awareness of adverse effects

Only 29 (14.5%) were aware of the adverse effects of SLT on pregnant mothers and fetuses, while more than half of the participants, 113 (56.5%), were aware of these for smoking. None of them could enlist/name any adverse effect. Women of younger age groups, educated above high school, and belonging to higher socioeconomic classes were aware of the adverse effects of smoking on mother and fetus (Table 2).

**Table 2:** Association of sociodemographic factors with awareness of adverse effects of antenatal smoking using Chi-square test (N=200) #Modified BG Prasad scale [15], * indicates p value ≤0.05

Variables	Aware	Unaware	Chi-square value	p- value
Age groups				0.001*
18 to 25 years	87(64.0)	49(36.0)	9.65	
26 to 33 years	26(40.6)	38(59.4)		
Education				<0.000*
Up till Middle school	40(32.8)	82(67.2)	71.57	
High school and above	73(93.6)	5(6.4)		
Occupation				0.069
Unemployed	106(58.5)	75(41.5)	3.30	
Employed	7(36.8)	12(63.2)		
Socio-economic status^#^				<0.000*
Class I and II	68(81.0)	16(19.0)	35.23	
Class III and IV	45(38.8)	71(61.2)		

None of the participants had considered quitting or received help or advice to quit SLT over the past 12 months. Merely two (1%) were aware of the national tobacco quit line number.

## Discussion

Current smokeless tobacco use among antenatal women of an urban resettlement colony of Delhi was found to be 14 (7%). The prevalence varied between 14.8% in Jharkhand and 30.9% in Maharashtra [13,16]. The prevalence of tobacco use was higher in these studies than in the present study. This might be because the present study is facility-based, and others are community-based. Also, the socio-demographic profile was different in those studies. The mean (SD) age of initiation of smokeless tobacco use in this study was 13.5 (1.9) years, which is less than that observed among antenatal women of Jharkhand, 17.1 (3.4) years [13]. More than half of Mishri users were initiated before the age of 10, as reported by Gupta et al. [17]. Parental imitation and the habit of purchasing smokeless tobacco for their mothers or elderly family members could have led to early smokeless tobacco exposure in these cases. Verma et al. found that concurrent use of smokeless tobacco or any tobacco product by the husband had influenced smokeless tobacco use in women [18]. In our study, we found that smokeless tobacco use was higher among migrant residents from Bihar and Uttar Pradesh engaged in daily wage labour. Ghutka was the most commonly used SLT product due to its availability, affordability, and taste, which were the most common reasons for its continued use. Studies have found misconceptions of medicinal benefits, including in pregnancy, social acceptance, and the role in culture and tradition, have all played a role in the current pattern of smokeless tobacco use [13].

Most, 143 (71.5%), of the women consumed the first smokeless tobacco of the day after 60 minutes of waking up, mirroring women’s behavior in Mumbai [4]. In our study, none initiated or changed the frequency of smokeless tobacco use. However, an increase and decrease in smokeless tobacco use were seen among antenatal women of Mumbai owing to reasons such as vomiting, relief of labor pain, and cravings associated with pregnancy [4].

Past smokers and users of smokeless tobacco were those who experimented with tobacco out of curiosity and discontinued it after being confronted by their immediate family members.

Significantly higher current smokeless tobacco use was seen among women aged >26 years, educated till middle school, employed, and belonging to the lower socioeconomic class. Analysis of NFHS 5 data reveals that tobacco use is higher among those belonging to the poor wealth quintile and those with fewer years of schooling [8].

Daily exposure to second-hand smoke at home was seen among 30% of women. Verma et al. found daily secondhand smoke exposure at home to be 40.2% [18]. Only 29 (14.5%) were aware that antenatal smokeless tobacco use has adverse effects, and the majority, 156 (78%), believed that exposure to second-hand smoke doesn't cause any serious illness. This highlights the hidden threat of antenatal smokeless tobacco use and how the SHS exposure is being overlooked. Awareness of the adverse effects of antenatal smokeless tobacco use was quite high (71.3%) among antenatal women of Sri Lanka [19].

None in this study had thought of quitting or received advice on quitting; however, Verma et al. reported that 36.3% of women thought of quitting tobacco during pregnancy, while only 3.8% of women were confronted by a treating physician on antenatal tobacco use [18]. This might be because of the high number of migrant populations in the study area and might be a missed opportunity for advice to quit tobacco use while visiting a health facility.

The results of this study have limited generalizability to community settings, as it has been conducted among antenatal women reporting to an urban health center. Also, caution might be taken while generalizing, as the present study was conducted in an urban resettlement colony of a metro city in an area that has a mostly migrant population. All the study participants were sensitized to the adverse effects of tobacco, especially smokeless tobacco and exposure to secondhand smoke. All the current smokeless tobacco users were counselled and referred to the nearest Tobacco Cessation Clinic.

## Conclusions

The findings of this study emphasize the high burden of antenatal tobacco exposure in the form of SLT and SHS along with poor awareness of the adverse effects of SLT use on mother and fetus. Raising awareness and dispelling myths about the supposed benefits of tobacco, especially SLT, is the need of the hour. Pregnancy creates a receptive environment for the expectant mother and her family to prioritize their health and well-being. The mandated four antenatal visits in India present a unique opportunity to introduce anti-tobacco initiatives in the family. Mandatory screening for tobacco use and integration of tobacco cessation services with antenatal care and capacity building of healthcare workers are crucial steps to end antenatal tobacco use which will have lasting benefits on the mother and the unborn child. Behavioral modification of smoking family members to reduce indoor smoking is essential to combat the hidden threat of SHS.

## Appendices

**Table 3:** Pretested questionnaire This questionnaire was adopted using the Global Adult Tobacco Survey (GATS) core questionnaire as reference [14]

Patient identification no.	Date of interview__/__/___	
Socio-economic and demographic data		
Name	Age	
Address	Contact number	
Religion	1.Hindu 2. Muslim 3.Others	
Occupation	1.Employed 2. Unemployed	
Educational status	1.Illiterate 2. Primary school. 3. Middle school. 4. High school. 5 Intermediate or post-high school 6. Graduate 7.Post-graduate	
Marital status	1. Never married 2. Currently married 3. Separated 4. Divorced 5. Widowed	
Total no. of family members	Total monthly family income	
Menstrual history	1.LMP 2. POG 3. EDD	
Obstetric history:	1.Gravida 2.Para 3. Abortion 4. Live	
Past Obstetric history:	1. Low birth weight baby 2.Bleeding before delivery (Antepartum hemorrhage) 3.Delivery before term (Pre term delivery)	
Past medical history:	1. Hypertension 2. Dyslipidemia 3.Diabetes mellitus 4. Others- (Please specify)	
Is current pregnancy high risk ?	1.Yes 2. No If yes, please specify	
Tobacco consumption history		
Do you currently smoke tobacco on a daily basis, less than daily, or not at all? DAILY ........................... 1 LESS THAN DAILY ...... 2 NOT AT ALL ................. 3		Do you currently use smokeless tobacco on a daily basis, less than daily, or not at all? DAILY ........................... 1 LESS THAN DAILY ...... 2 NOT AT ALL ................. 3
In the past, have you smoked tobacco on a daily basis, less than daily, or not at all? DAILY ........................... 1 LESS THAN DAILY ...... 2 NOT AT ALL ................. 3		In the past, have you used smokeless tobacco on a daily basis, less than daily, or not at all? DAILY ........................... 1 LESS THAN DAILY ...... 2 NOT AT ALL ................. 3
How old were you when you first tried smoking tobacco, even once? ……………….. How many years ago did you first try smoking tobacco, even once? …………………		How old were you when you first tried using smokeless tobacco, even once? …………… How many years ago did you first try smokeless tobacco, even once? …………………
How old were you when you first started smoking tobacco daily? ……………………		How old were you when you first started using smokeless tobacco daily? ……………………
How many years ago did you first start smoking tobacco daily? ……………………..		How many years ago did you first start using smokeless tobacco daily? ……………………..
Why do you smoke tobacco?		Why do you use smokeless tobacco?
On average, how many times do you use the following products? PER DAY PER WEEK Cigarettes Bidi Cigar Hookah Others (Specify)		On average, how many times do you use the following products? PER DAY PER WEEK Snuff, by mouth Snuff, by nose? Chewing tobacco? Khaini, tooth paste, mishri Betel quid with tobacco? Others (Specify)?
How soon after you wake up do you usually have your first smoke? WITHIN 5 MINUTES .................. 1 6 TO 30 MINUTES..................... 2 31 TO 60 MINUTES .................. 3 MORE THAN 60 MINUTES ....... 4		How soon after you wake up do you usually use smokeless tobacco for the first time? WITHIN 5 MINUTES .................. 1 6 TO 30 MINUTES..................... 2 31 TO 60 MINUTES .................. 3 MORE THAN 60 MINUTES ....... 4
Do you believe smoking tobacco has adverse health effects on pregnant mother? Yes…….1 No………2		Do you believe using smokeless tobacco has adverse health effects on pregnant mother? Yes…….1 No………2
If yes, which of these do you believe are the adverse effects on the mother? 1.Preterm labour 2.Placenta previa 3.Ectopic pregnancy 4.Others (Specify)		If yes, which of these do you believe are the adverse effects on the mother? 1.Preterm labour 2.Placenta previa 3.Ectopic pregnancy 4.Others (Specify)
Do you believe smoking tobacco has adverse health effects on the fetus? Yes…….1 No………2		Do you believe using smokeless tobacco has adverse health effects on the fetus? Yes…….1 No………2
If yes, which of these do you believe are the adverse effects on the fetus? Intrauterine growth restriction Low birth weight Congenital birth defects Still birth Others (Specify)		If yes, which of these do you believe are the adverse effects on the fetus? Intrauterine growth restriction Low birth weight Congenital birth defects Still birth Others (Specify)
During the past 12 months, have you tried to stop smoking tobacco? Yes…….1 No……..2		During the past 12 months, have you tried to stop using smokeless tobacco? Yes…….1 No……..2
If yes, why? Concern for your personal health That society disapproves of smoking tobacco? The price of tobacco products? Smoking tobacco is/was not allowed in your home? Smoking tobacco restrictions at work or public places? Wanting to set a good example for children? Close friends and family disapprove of you smoking tobacco? Concern for adverse health effects in pregnancy Others (Specify)		If Yes, why? Concern for your personal health? That society disapproves of using smokeless tobacco? The price of smokeless tobacco products? Smokeless tobacco use is/was not allowed in your home? Smokeless tobacco restrictions at work or public places? Wanting to set a good example for children? Close friends and family disapprove of your using smokeless tobacco? Concern for adverse health effects in pregnancy Others (Specify)
Have you ever received help or advice to help you stop smoking? Yes……1 No…….2		Have you ever received help or advice to help you stop using smokeless tobacco products? Yes……1 No…….2
If yes, from whom? a. Counseling, including at a smoking cessation clinic ? b. Family member or friend? c. Traditional medicines? d. A quit line or a smoking telephone support line? Others (Specify)		If yes, from whom? a. Counseling, including at a tobacco cessation clinic? b. Family member or friend? c.Traditional medicines? d. A quit line or telephone support line? Others (Specify)
Thinking about the last time you tried to quit, how long did you stop smoking tobacco? MONTHS .......................................... 1 WEEKS ............................................. 2 DAYS ................................................ 3 LESS THAN 1 DAY (24 HOURS) .....		Thinking about the last time you tried to quit, how long did you stop using smokeless tobacco? MONTHS .......................................... 1 WEEKS ............................................. 2 DAYS ................................................ 3 LESS THAN 1 DAY (24 HOURS) ..... 4
How long has it been since you stopped smoking? YEARS .......................... 1 MONTHS ...................... 2 WEEKS ......................... 3 DAYS ............................ 4 LESS THAN 1 DAY ...... 5		How long has it been since you stopped using smokeless tobacco products? YEARS .......................... 1 MONTHS ...................... 2 WEEKS ......................... 3 DAYS ............................ 4 LESS THAN 1 DAY ...... 5
Are you aware of national tobacco quit line number? Yes………1 No……….2		Are you aware of national tobacco quit line number? Yes………1 No……….2
Secondhand tobacco smoke exposure		
1.Which of the following best describes the rules about smoking inside of your home? ALLOWED .............................................. 1 NOT ALLOWED, BUT EXCEPTIONS .... 2 NEVER ALLOWED ................................. 3 NO RULES ............................................. 4		
2.How often does anyone smoke inside your home? DAILY .................................. 1 WEEKLY .............................. 2 MONTHLY ........................... 3 LESS THAN MONTHLY ...... 4 NEVER ................................ 5 DON’T KNOW ...................... 6		
Answer the questions from 3 to 9 with the following code: YES ......................... 1 NO .......................... 2 DON’T KNOW ......... 3		
3.During the past 30 days, did anyone smoke in indoor areas where you work?		
4.Did anyone smoke inside of any government buildings or government offices that you visited in the past days?		
5.Did anyone smoke inside of any health care facilities that you visited in the past 30 days?		
6.Did anyone smoke inside of any restaurants that you visited in the past 30 days?		
7.Did anyone smoke inside of any public transportation that you used in the past 30 days?		
8.Did anyone smoke inside of any school buildings or universities that you visited in the past 30 days?		
9.Based on what you know or believe, does breathing other people’s smoke cause serious illness in non-smokers?		
